# Early bone growth on the surface of titanium implants in rat femur is enhanced by an amorphous diamond coating

**DOI:** 10.3109/17453674.2011.579522

**Published:** 2011-09-02

**Authors:** Jarkko JP Jaatinen, Rami K Korhonen, Alpo Pelttari, Heikki J Helminen, Hannu Korhonen, Reijo Lappalainen, Heikki Kröger

**Affiliations:** ^1^Department of Orthopedics, Bone and Cartilage Research Center, Kuopio University Hospital; ^2^Department of Applied Physics; ^3^BioMater Center, University of Eastern Finland; ^4^Department of Anatomy, Institute of Biomedicine, University of Eastern Finland, Kuopio, Finland

## Abstract

**Background and purpose:**

Amorphous diamond (AD) is a durable and compatible biomaterial for joint prostheses. Knowledge regarding bone growth on AD-coated implants and their early-stage osseointegration is poor. We investigated bone growth on AD-coated cementless intramedullary implants implanted in rats. Titanium was chosen as a reference due to its well-known performance.

**Materials and methods:**

We placed AD-coated and non-coated titanium implants (R_a_ **≈** 0.2 μm) into the femoral bone marrow of 25 rats. The animals were divided in 2 groups according to implant coating and they were killed after 4 or 12 weeks. The osseointegration of the implants was examined from hard tissue specimens by measuring the new bone formation on their surface.

**Results:**

4 weeks after the operation, the thickness of new bone in the AD-coated group was greater than that in the non-coated group (15.3 (SD 7.1) μm vs. 7.6 (SD 6.0) μm). 12 weeks after the operation, the thickness of new bone was similar in the non-coated group and in the AD-coated group.

**Interpretation:**

We conclude that AD coating of femoral implants can enhance bone ongrowth in rats in the acute, early stage after the operation and might be an improvement over earlier coatings.

One of the important factors preventing loosening of joint prosthesis is early fixation, i.e. firm attachment of the prosthesis to the bone. The mechanical properties of modern bone cements correspond well with those of bone, and the survival of cemented implants is good (Aamodt et al. 2004). However, cementless prostheses have been suggested to have minimal stress shielding (Ellison et al. 2009) and even superior survival rate (Moreland and Moreno 2001, Emerson et al. 2002, Sorrells et al. 2004, Hooper et al. 2009, Yamada et al. 2009).

Because bone attachment is much more durable and stable than fibrous attachment, the best type of prosthesis attachment is achieved with new bone contact and not by scarring. The formation of new bone is most active in the early postoperative stage. On the other hand, the bone loss is at its highest level in the acute stage. After this stage, the velocity of bone loss equals that of normal age-induced osteoporosis (Venesmaa et al. 2000, 2001, 2003). One way to influence periprosthetic bone loss and related bone density changes is use of better coatings for early-stage osseointegration (Schopper et al. 2005). Hydroxyapatite coating has a bone growth-inducing effect (Santori et al. 2001). Fluorapatite, which releases less ions to the surrounding tissue than hydroxyapatite, also enhances osseointegration (Bhadang and Gross 2004). In a study by Guglielmotti et al. (1999), implants coated with diamond-like carbon (DLC) were placed in the tibias of Wistar rats and were found to osseointegrate better than non-coated titanium implants.

Amorphous diamond (AD), one of the DLC coatings, is a durable, versatile and highly bio-compatible biomaterial that is scratch-resistant and forms a strong attachment to bone cement (Santavirta et al. 1999, Toyras et al. 2005, Lappalainen and Selenius 2008). However, our knowledge regarding bone growth on the surface of AD at different follow-up times is still poor. We examined bone growth on AD-coated femoral implants in rats 4 and 12 weeks postoperatively. We hypothesized that AD enhances osseointegration in both of the observation points mentioned above. The reason for the study was investigation of possible clinical applications as a prosthesis coating for cementless fixation.

## Material and methods

### Implants

Both non-coated implants and AD-coated implants (pure AD, sp^3^ diamond bonding: >70%) were made of titanium rod, 1.9 mm in diameter and 18 mm in length. The titanium was 99.6% pure annealed rod (Goodfellow Metals, Huntingdon, England). Preliminary implantation tests using dead rats indicated that these implant dimensions guaranteed a sufficient press-fit tightness through the rat femur.

**Figure 1.**
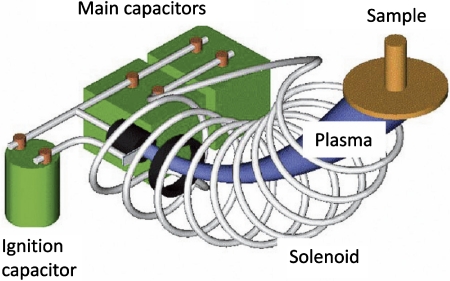
The AD coating was attached to the titanium implants with the filtered, pulsed plasma arc-discharge method (Lappalainen and Santavirta 2005).

The AD coating was deposited on the entire length of the implants by filtered, pulsed plasma arc-discharge method (Lappalainen and Santavirta 2005) at the University of Eastern Finland (Figure 1). The surfaces of polished titanium rods were cleaned with an Ar^+^-ion beam in a vacuum of 10 mPa. The carbon plasma was produced in a vacuum (300 μPa) by letting the capacitor discharge between a graphite anode and cathode. The plasma pulse created was directed to the samples by electric coils at room temperature. To further enhance the adhesion, high-energy plasma beam was used (140 keV) at the beginning, but the main part of the AD coating was deposited with low energy, only a few eV, to achieve a high proportion of sp^3^ bonds. The average roughness (R_a_ ) of the surface of the implants was ≈ 0.2 μm (Santavirta et al. 1999, Lappalainen and Santavirta 2005, Lappalainen and Selenius 2008). The roughness was determined for uncoated rods (polished plain titanium) and for coated rods using a Mitutoyo Surftest SJ301 roughness tester (Mitutoyo Corp., Kawasaki, Japan), and it was the same for both of them. The thickness of the AD coating was about 500 nm.

### Operation

**Figure 2.**
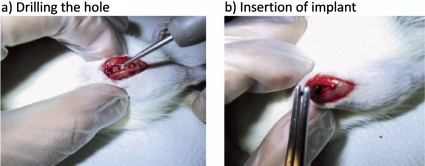
The operation. Drilling of the hole between the femoral condyles into the bone marrow cavity (a) and insertion of the implant (b). See text for details.

16-week-old Wistar rats were used in the tests. They were anesthesized by inhalation of halothane. An incision of approximately 2 cm was made to the right hind leg of the rats, on the lateral side of the knee. The knee joint was opened by making a 1–1.5-cm incision on the lateral side of the patella. The patella—together with the ligament—was then lifted to the medial side, thus revealing the intercondylar space. A hole was drilled into the bone marrow cavity between the condyles using a thin skull drill. Then the primary drill hole was widened with a 1.9-mm drill and the drilling was continued in the bone marrow cavity lengthwise to the bone. The titanium implant, either AD-coated or non-coated, was then placed in the bone marrow cavity (Figure 2). The implant was pushed deep enough to make sure that the tip of the implant was not sticking out from the joint surface. The drilled hole was left open. During the implantation, the knee joint was lavaged with physiological saline to keep the surface of the joint wet and to rinse out the debris such as bone dust from the drilling. The joint was closed with biodegradable sutures. Buprenorphine was used as pain medication in weight-related doses of 0.03 mg/kg. No postoperative antibiotic treatment was given. All rats used the operated leg naturally after surgery.

The procedure was conducted under the permission of the Animal Care Committee of the Provincial Government of Eastern Finland (ISLH-2002-02083/Ym-23). The operations were performed in the facilities of the National Center for Laboratory Animals of the University of Eastern Finland, Kuopio, Finland.

### Sample preparation

The rats were first divided into 2 groups according to the coating, and these groups were further divided into 4- and 12-week observation groups. One group consisted of 7 rats (non-coated, 4-week observation) and the other groups consisted of 6 rats. After killing the rats according to the guidelines of the National Center for Laboratory Animals, the femurs were prepared, dehydrated in alcohol, and embedded in methylmethacrylate. The Macro Exakt 310 CP saw and the Exakt 400 CS grinder (EXAKT Technologies Inc., Oklahoma City, OK) were used to prepare thin slices (transverse to the bone) for light microscopy. The final thin slices (∼20 μm in thickness) were dyed with toluidine blue.

### Analysis of the samples

**Figure 3.**
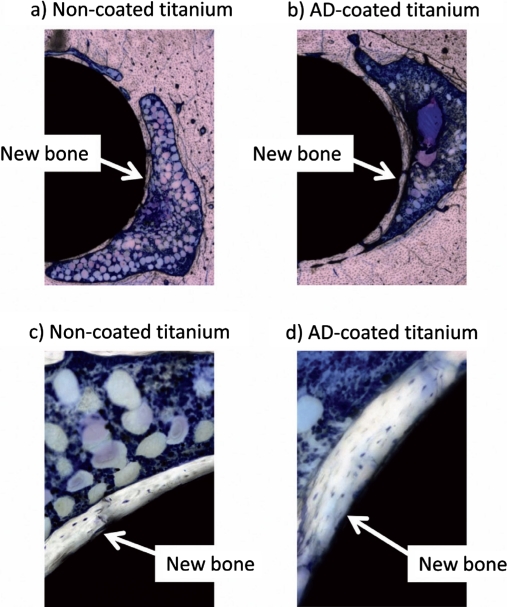
Representative examples of light microscopic images of non-coated (a, c) and AD-coated (b,d) titanium implants in rat femurs 12 weeks after the surgical procedure. In this example, the new bone layer is much thicker on the AD-coated implant surface than on the non-coated implant surface. Toluidine blue was used for staining. It is noteworthy that a fibrous capsule was not visible on the AD-coated implants. 2 × magnification (a, b) and 10 × magnification (c, d).

Light microscopic images were acquired from the thin sections and AnalySIS software (Soft Imaging System GmbH, Münster, Germany) was used for image analysis. The percentage of new bone attached to the implant surface and the average thickness of the new bone layer were measured (Figure 3). The area of the primary press-fit contact of the implant and bone was excluded from the analysis. Thus, we analyzed only new bone (Miettinen et al. 2009). 10 thin slices were processed and analyzed from 1 sample, and showed consistent analysis parameters throughout the length of the implant (see below). Finally, 3 thin slices were analyzed from the rest of the samples: 1 from the proximal and distal ends of the diaphysis and 1 from the middle part.

The percentage of new bone in contact with the implant surface was calculated as a ratio of the area of contact of the implant with new bone and the perimeter of the implant, excluding the primary press-fit contact of the implant and bone. The average thickness of the new bone layer was calculated by segmenting the outer surface of the new bone and the implant surface, and by calculating the average distance between these surfaces. The average of both of the parameters above was first calculated from 3 slices of each sample. These average values were then used to calculate the mean values of the analysis parameters for each group.

### Statistics

Mann-Whitney U test in SPSS statistical software (SPSS Inc., Chicago, USA) was used for the comparison between the AD-coated and the non-coated groups. Furthermore, the 4-week groups with each coating were compared with the 12-week groups with the same coating.

## Results

**Figure 4.**
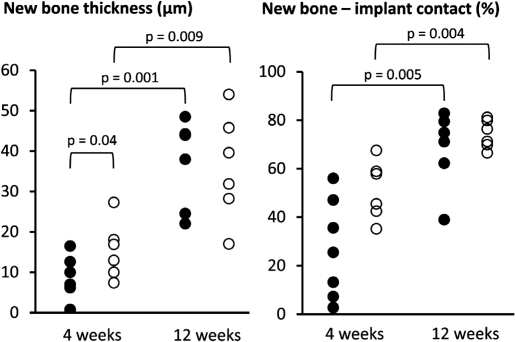
Thickness of new bone layer on the implant surface (left) and percentage of implant surface covered with new bone (right) for non-coated (•) and AD-coated (○) titanium implants 4 and 12 weeks after surgical operation. Each data point is shown, and statistically significant differences between the groups and time points are indicated.

4 weeks after the operation, the new bone layer on the AD-coated implants was thicker than that on the plain titanium implants (Figure 4) (p = 0.04). The thickness of new bone on the AD-coated and non-coated implants was similar 12 weeks after the operation (Figure 4).

4 weeks after the operation, about half of the surface of the AD-coated implants was covered with new bone, while only ∼25% of the non-coated implants was covered with new bone (Figure 4). 12 weeks after the operation, the fractions of new bone covering the implant surfaces were similar in both groups (Figure 4). There were no statistically significant differences in the fractions of new bone on the implants between the non-coated and AD-coated groups.

Both of the parameters analyzed were significantly greater 12 weeks after the operation in both groups (Figure 4) (p = 0.001 to p = 0.009).

## Discussion

The results indicate that early bone growth (4 weeks after the operation) on the surface of titanium implants in rat femur was enhanced by the use of an AD coating. On the other hand, new bone thickness and bone-implant contact were similar in AD-coated and non-coated implants 12 weeks after the operation.

The results are in line with earlier results gathered from DLC coating research. For example, in the study by Guglielmotti et al. (1999), different kinds of implants (titanium, zirconium, aluminium, and DLC-coated zirconium) were placed in the tibias of Wistar-rats for 30 days. The DLC coatings became better osseointegrated than non-coated titanium implants.

Because the number of animals in our study was small (n = 25) and only one of the variables was statistically significantly different between the non-coated and AD-coated groups, and only at one time point, it could be argued that the results may be a random phenomenon. Even so, in principle AD and titanium surfaces are different. AD surfaces are stable, and wear- and corrosion-resistant at all times. However, a thin TiO_2_ oxide layer is spontaneously formed on the surface of titanium. Movement at the interface may damage this protective oxide, and as a result this layer is rapidly replaced by a repassivation layer. Thus, AD may provide faster bone formation due to stable surface properties. The non-existent fibrous tissue around AD-coated rods further supports this supposition.

12 weeks after the operation, the difference between the groups had disappeared. We speculate that the AD-coated implants may induce bone remodeling faster at the end of the acute phase. On the other hand, since the bone layer covering the surface of the implant was thicker around AD-coated implants than around non-coated implants 4 weeks after the implantation, the surface properties of the coated implants after that time point may not induce bone formation as much as those of plain titanium implants. However, titanium implants, which have a thinner layer of bone on their surface 4 weeks after the operation, may provoke better bone formation after that time point, while bone remodeling may be induced at a slower pace. No bone labels were used in this study; thus, the bone formation rates and modeling/remodeling at different time points remain speculative.

Biomechanical tests, such as pull-out tests, would have provided more information about the stability of the interface between the implant surface and new bone. It has been suggested that both the percentage of the implant surface covered by bone and bone thickness may improve osseointegration and anchoring of implants, characterized by mechanical testing (Brånemark et al. 1997, Ferris et al. 1999, Lan et al. 2007, Xiao et al. 2010). Here, we can only speculate that a thicker layer of new bone on the implant surface and a larger surface area of the implant covered by bone may improve the anchoring of the rods in the femur.

It is important to acknowledge that neither the bone marrow cavities of the femurs nor the new bone formations were symmetric or regular in shape. This source of error was minimized by taking 3 samples from each animal for analysis: from the proximal and distal ends of the diaphysis and from the middle part, halfway between the 2 former. In the analysis, a mean value of these 3 slices was used.

Earlier results on the wear and stability of the implants coated with the same technique as we used showed that the AD-coated implants can withstand much greater forces than the rods in this study (Santavirta et al. 1999, Lappalainen et al. 2003, Lappalainen and Santavirta 2005). This suggests that the AD coatings remained stable and intact during the entire experimental protocol of our study.

The effect of making the AD coating porous and the effect of the roughness of the AD coating on the osseointegration should be studied. In this study, both titanium and AD-coated titanium surfaces had the same roughness, in order to study the effect of the materials. It can be assumed that porous or roughened surfaces increase the surface adhesion between the bone and the implant, and enhance the attachment (Yamaguchi et al. 2000). A question will arise about whether the entire prosthesis should be coated in clinical practice or whether the coating on the metaphyseal region of the prosthesis would suffice (Santori et al. 2001). In the present study, the entire implants were AD-coated and bone formation was evenly distributed throughout the length of the implant.

Our study focused entirely on the performance of AD as a coating material on the adhesion surfaces of the implants. In previous experiments, AD has been used as a coating on the sliding joint surfaces of prostheses (Lappalainen and Selenius 2008). Its resistance to scratching is excellent (Santavirta et al. 1999, Lappalainen and Santavirta 2005, Lappalainen and Selenius 2008). The scratching of metallic implants may cause increased friction between sliding joint surfaces, and metallosis. Both of these phenomena increase the risk of loosening of the prosthesis. Thus, the idea of using AD coating not only for the adhesion surfaces but also on the sliding joint surfaces of the prosthesis, and as a protection against corrosion, should be considered.

We conclude that AD coating can enhance osseointegration of implants in the acute, early stage after surgery.
